# Effective Methods Based on Distinct Learning Principles for the Analysis of Hyperspectral Images to Detect Black Sigatoka Disease

**DOI:** 10.3390/plants11192581

**Published:** 2022-09-30

**Authors:** Jorge Ugarte Fajardo, María Maridueña-Zavala, Juan Cevallos-Cevallos, Daniel Ochoa Donoso

**Affiliations:** 1 Facultad de Ingeniería Industrial, Universidad de Guayaquil, Guayaquil 090601, Ecuador; 2 Centro de Investigaciones Biotecnológicas del Ecuador (CIBE), ESPOL Polytechnic University, Escuela Superior Politécnica del Litoral, ESPOL, Guayaquil 090902, Ecuador; 3 Facultad de Ciencias de la Vida (FCV), ESPOL Polytechnic University, Escuela Superior Politécnica del Litoral, ESPOL, Guayaquil 090902, Ecuador; 4 Facultad de Ingeniería Eléctrica y Computación (FIEC), ESPOL Polytechnic University, Escuela Superior Politécnica del Litoral, ESPOL, Guayaquil 0909022, Ecuador

**Keywords:** black sigatoka, deep learning, hyperspectral imaging, machine learning, plant disease

## Abstract

Current chemical methods used to control plant diseases cause a negative impact on the environment and increase production costs. Accurate and early detection is vital for designing effective protection strategies for crops. We evaluate advanced distributed edge intelligence techniques with distinct learning principles for early black sigatoka disease detection using hyperspectral imaging. We discuss the learning features of the techniques used, which will help researchers improve their understanding of the required data conditions and identify a method suitable for their research needs. A set of hyperspectral images of banana leaves inoculated with a conidial suspension of black sigatoka fungus (*Pseudocercospora fijiensis*) was used to train and validate machine learning models. Support vector machine (SVM), multilayer perceptron (MLP), neural networks, N-way partial least square–discriminant analysis (NPLS-DA), and partial least square–penalized logistic regression (PLS-PLR) were selected due to their high predictive power. The metrics of AUC, precision, sensitivity, prediction, and F1 were used for the models’ evaluation. The experimental results show that the PLS-PLR, SVM, and MLP models allow for the successful detection of black sigatoka disease with high accuracy, which positions them as robust and highly reliable HSI classification methods for the early detection of plant disease and can be used to assess chemical and biological control of phytopathogens.

## 1. Introduction

Black sigatoka (BLSD) is the most destructive leaf disease of the banana, affecting a wide range of cultivars and causing production losses that could reach 85%. Up to 50 annual fungicide aerial application cycles are needed to control BLSD, which accounts for 15% to 27% of the total annual production costs [1].

This disease is caused by the pathogenic fungus *Pseudocercospora fijiensis*, which destroys the photosynthetic leaf tissue affecting chlorophyll production [2]. Fungal spores colonize the leaf mesophyll through the stomata affecting photosynthesis, which causes damage to the leaf [3]. It is well-known that leaf physiological changes produce reflectance variations in the VIS, NIR, and MIR regions of the electromagnetic spectrum.

In recent years, hyperspectral technology has received a great boost due to the advent of smart agricultural and food-industry practices. Hyperspectral sensors enable fast, accurate, and reliable recognition of the plant characteristics that a human eye is not capable of observing, making them a promising non-destructive alternative for the field assessment of plants’ health and food quality assurance [4]. For effective data processing, centralized computing system architectures have been used in a variety of applications for agriculture. On the other hand, an edge computing system ensures real-time processing at the edge of the network, specifically, an unmanned aerial vehicle (UAV)-aided edge computing system where a UAV serves as a node that executes various computational tasks [5].

**Figure 1:**
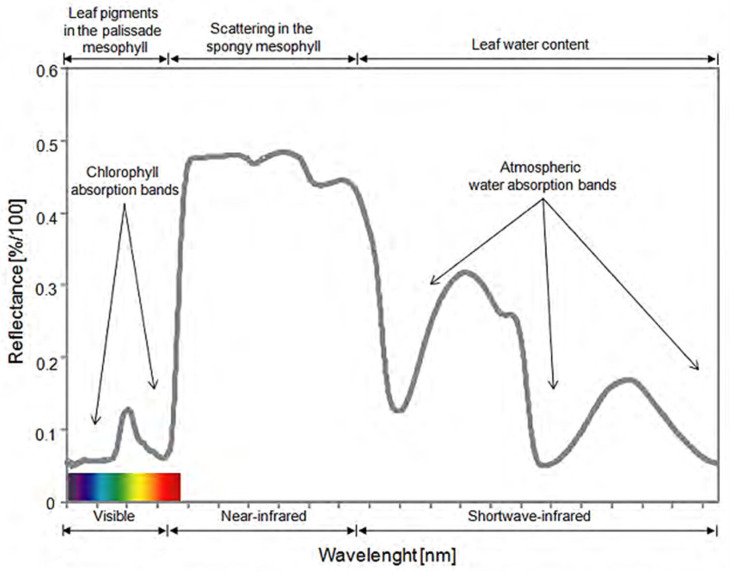
Spectral signature of healthy vegetation [12].

For instance, drone-based crop imaging makes it possible to perceive plant damages caused by pathogens by assessing the changes in the concentration of leaf pigments [6,7] that manifest themselves in the spectral signature (Figure 1).

The spectral signature is generated from hyperspectral data and represents the reflectance produced by interactions between radiant energy and the biochemical characteristics of leaves [8]. Thus, the leaf cell structure determines the reflectance in the near-infrared region (NIR 750–1350 nm), while photosynthetic pigments change the reflectance in the visible region (380–780 nm), and water content defines the reflectance levels in the mid-infrared region (1350–2500 nm) [9]. Therefore, physiological changes, caused by pathogens, produce changes in the spectral signature of plants [10,11].

### Plant Disease Detection Using Hyperspectral Imaging

Hyperspectral imaging-based non-destructive methods for the characterization of plant diseases have been developed. There are various Advanced machine learning (ML) techniques for Hyperspectral image analysis, such as support vector machine (SVM), artificial neural network (ANN), partial least square–discriminant analysis (PLS-DA), linear discriminant analysis (LDA), and random forests (RF). A compilation of the significant contributions to the applications of machine learning methods in plant disease detection was presented by Lowe [13] and Liakos [14].

Huang [15] used the relationship between the disease index (DI) and the photochemical reflectance index (PRI) using simple linear regression to quantify wheat yellow rust, reaching a coefficient of determination (R2) of up to 0.97. Rumpf [16] presented a proposal for the early detection of beet diseases with SVM models generated from vegetation indices. Although this model achieved a low classification rate in early disease stages, it exceeded 95% accuracy in the leaves showing infection areas greater than 6%. This study also evaluated decision trees and artificial neural networks, but the classification error was higher than in the SVM models. In a similar study, A. Mahlein [12] used hyperspectral imaging to identify three beet leaf diseases: Cercospora leaf spot (CLS), powdery mildew (PM), and beet rust (SBR). The study combined several vegetation indices and built SVM models to classify healthy (non-inoculated) and inoculated leaves with an accuracy between 93% and 97%. For the quantification of the diseases, the spectral angle mapper (SAM) method was applied to achieve the Kappa classification coefficients of 0.98, 0.95, and 0.56. Zhu [17] used the successive projection algorithm (SPA) for the preselection of a small number of representative wavelengths in Tobacco mosaic virus infection. For the prediction of the disease SVM, backpropagation neural network (BPNN), extreme learning machine (ELM), least square–support vector machine (LS-SVM), PLS-DA, linear discriminant analysis (LDA), and RF methods were used to achieve a prediction accuracy over 85%. Ugarte [18] used the partial least square–penalized logistic regression (PLS-PLR) and hyperspectral biplot (HS-Biplot) techniques for the early detection of black sigatoka disease (BLSD) using hyperspectral images, achieving 98% accuracy. The relationships between groups of healthy and diseased leaves and their relationships with the wavelengths of the VIS and NIR regions were graphically represented using HS-Biplot.

Machine learning and deep learning techniques have been used as tools for endophyte bioprospecting and the biological control of phytopathogens [19]. Sanchez-Azofeifa [20] explored the relationship between endophyte colonization, leaf traits such as specific leaf weight, water content, polyphenols, chlorophyll, and carotenoid concentration in Coccoloba cereifera leaves, which, in turn, can be detected in the hyperspectral reflectance spectra. Specifically, endophyte richness affects the reflectance in the visible and near-infrared wavelengths. Therefore, changes in the spectral signature can be used to estimate endophyte abundance. Vladimir Vujanovic [21] used FTIR spectroscopy and hyperspectral imaging to assess the kernels of AC Avonlea durum wheat–fungal endophyte environment interaction. The acquired differentiation in the coleorhiza composition of *Triticum durum* between ambient and drought stress conditions highlights the use of FTIR spectroscopy to understand the early plant–endophyte interaction based on molecular changes in the coleorhiza during the kernel germination stage.

Numerous machine learning methods for the detection of plant diseases have been exploited; however, hyperspectral imaging uses high-resolution reflectance information over a large range of the electromagnetic spectrum and thus has the potential for identifying subtle changes in infected leaves before any visible symptoms of infection appear. We investigated the potentiality of the early detection of black sigatoka disease using hyperspectral imaging, combined with machine learning classifiers. In this work, we evaluated four machine learning techniques that use distinct mathematical principles to approximate an underlying prediction function that can be used to solve several research problems with different hyperspectral data conditions.

## 2. Materials and Methods

### 2.1. Plant Material

**Figure 2:**
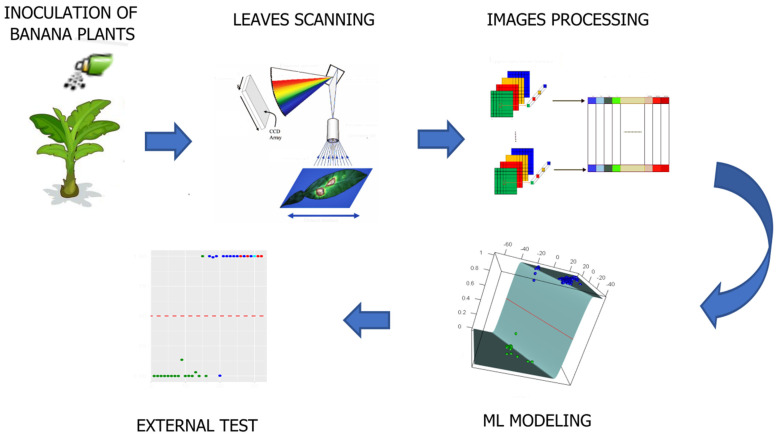
Schematic diagram of the research process to detect black sigatoka disease using hyperspectral images.

Healthy and BLSD-infected Banana plants (Musa acuminata, AAA Group, Cavendish subgroup, cultivar ‘Williams’) were obtained as described by Ugarte et al. [18]. For this study, 3–4-month-old plants were kept in a greenhouse at 28 °C in 70% relative humidity and 12 h of natural light and were watered every 48 h. In total, 16 plants were randomly selected, of which 10 were inoculated with *P. fijiensis*, and 6 were mock-inoculated with distilled autoclaved water. The inoculation was carried out using a conidial suspension prepared as suggested by Gbongue [22] and banana leaves were spray-inoculated with the concentrated conidia suspension using an aerograph atomizer (Gerensa, Guayaquil, Ecuador). The disease symptoms were monitored using the severity scale shown in Table 1, as suggested by Fouré [23]. Figure 2 shows a schematic diagram of the process of this research.

### 2.2. Data

A set of images of banana leaves infected and non-infected with BLSD were obtained with an ImSpector V10E hyperspectral scanner and subjected to a pixel binning process followed by spectral and radiometric calibration, achieving, for each image, a hyperspectral (HIS) cube of 205 rows, 198 columns, and a spectral dimension of 520 wavelengths. The process is described in detail in Ugarte et al. [18].

The training dataset of 104 leaf images included 16 uninfected and 88 infected with initial levels of infection (16 presymptomatic, 54 at severity level 1, and 18 at severity level 2), whereas the test dataset consisted of 32 leaf images, 16 uninfected and 16 infected with the following infection severity levels: 1 presymptomatic, 11 severity level 1, 4 severity level 2.

### 2.3. Preprocessing

Each HSI cube was normalized using the standard normal variate (SNV) to correct for the scale differences in the reflectance measurements produced by variations in the distance to the target and in the light source. Dimensionality reduction was applied to each cube by calculating the mean of the reflectance values for each wavelength, resulting in a matrix of 104 rows (one per image) and 520 columns.

### 2.4. Model Building

The NPLS-DA, PLS-PLR, SVM, and MLP neural network methods were selected for their high and recognized predictive ability, as demonstrated in numerous studies. Furthermore, they are based on distinct statistical learning principles for finding predictive functions based on the analyzed data.

The classification models were trained using the data described in previous paragraphs. The results were evaluated using confusion matrices and prediction metrics such as precision, sensitivity, accuracy, and F1. The receiver operating characteristic (ROC) curves and the area under the ROC curve (AUC) were used to evaluate the suitability of the models.

The PLS-PLR and NPLS-DA models were implemented using the R language, while in the SVM and neural network models, the python Scikit-learn and TensorFlow libraries were used. The source programs are available at the following link: https://github.com/JUG2019/Sigatoka-detect (accessed on 21 August 2022).

To evaluate the effectiveness of the models fitted to the training dataset, a test dataset was used to predict the presence of the disease. The new dataset included images of 16 uninfected and 16 infected leaves in the presymptomatic stage and severity levels 1 and 2.

The NPLS-DA algorithm is based on the PLS1 algorithm, but the answer is binary (Ouertani et al. 2014). In order to generate the NPLS-DA model, a third-order tensor $\underline{X}$ was designed using a matrix-making method proposed by Folch-Fortuny [24]. In each HS cube of dimensions 205 rows, 198 columns, and 520 wavelengths, 5 features (mean, standard deviation, symmetry, kurtosis, and fifth moment) were calculated at each wavelength, obtaining a tensor of dimensions 104 × 5 × 520. This new data cube was used for the training and testing of the NPLS-DA model.

The construction of a calibration model with second-order data (each observation is a matrix) can be performed by unfolding the three-way data in such a way that a first-order tensor is obtained for each element of the sample. In this case, three-way data analysis methods can be used [25]. Thus, a three-way array of independent variables is converted into a two-dimensional unfolded matrix. The matrix is decomposed in a triad similar to the PARAFAC model [26], but in this case, it is adjusted following the PLS philosophy. The triad is formed by the scores vector t and two weights vectors (w), one of the second mode ***W^J^*** (*J* × 1) and one of the third mode ***W^K^*** (*K* × 1) with a length equaling 1 [27]. The model can be written: $X=T{\left(W^{K}⊗W^{J}\right)}^{T}+E_{x}$, where ***X*** is the mode-1 unfolded third-order tensor, ***T*** is the score matrix, and ***y*** and ***W*** are the weight matrices. The probability of infection is calculated by *logit*($\hat{y})=Tb$, where ***b*** is the regression coefficient of $\hat{y}$ on score ***T***.

PLS-PLR is a classification method with high predictive power that includes some features that allow for solving some problems that occur in hyperspectral data such as multicollinearity, a reduction in bias, and over-fitting. In this method, the linear regression relating the response to the PLS components in the original algorithm is replaced by a penalized logistic regression, thus achieving a better fit to the binary response. The final model is the logistic regression of ***y*** on the retained PLS components, $logit\left(\hat{y}\right)=c_{0}1+Tc=c_{0}1+XWc=c_{0}1+Xb,$ where $\hat{y}$ is the vector of the estimated probabilities of occurrence, c is the coefficient of the regression on a component, and W and b are the coefficients on the observed variables.

To obtain the PLS-PLR model with the best fit, the penalty parameter Ridge (λ) was carefully chosen using goodness-of-fit metrics such as the deviancy difference (88.488), MacFadden’s R^2^ (0.991), Cox and Snell R^2^ (0.573), and Nagelkerke R^2^ (0.994). The model was validated using the leave-one-out cross-validation (LOOCV) method, and the external validation was carried out using a test dataset consisting of 32 images [18].

SVM is a supervised method widely used in hyperspectral image classification because of its strength as a linear classifier either with separable original data (original space) or with transformed data for the case where the original data are not separable. The SVM algorithm searches for a hyperplane with the lowest structural risk by maximizing the margin of the separation of the two classes (maximum margin classifier). For this purpose, 2 parallel hyperplanes, H1 and H2, equidistant from the central hyperplane on which the support vectors are located, are initially considered. The maximum distance between H1 and H2 is obtained by minimizing $\frac{1}{2}{\left\|w\right\|}_{2}$ subject to the constraint $y_{i}(w\cdot x_{i}+b)-1\geq \;0$ for all i, which determines the location of the points *x_i_* in each class. ${\left\|w\right\|}_{2}$ is the Euclidean norm of the coefficient vector of the hyperplane. The solution to this problem is obtained by applying quadratic programming techniques [28].

Since our model is a binary classifier for infected and non-infected leaves, SVM classifiers with linear, polynomial, and radial basis function kernels were evaluated, and the first two were selected for the best prediction results. The kernel function transforms the input data by projecting them to a higher dimensional feature space (the Hilbert space) [29].

Initially, several linear models were evaluated by changing the C regularization hyperparameter between 1 and 50. With the default C value equal to 1, the best prediction results were obtained.

Then, the SVM models with second- and third-degree polynomial kernels were evaluated by changing the C hyperparameter from 1 to 100. The best results were obtained with a second-degree polynomial kernel and the C hyperparameter equaling 91.

In the cost function, the C hyperparameter regulates the permissibility of errors. If the value of C is too large, the optimal margin width will be narrower, and the resulting model will fit the data fairly well with a reduced bias, but the variance will increase, and therefore, the risk of overfitting will also increase. On the other hand, if C is too small, the optimal margin will be wider, allowing a higher number of elements within the margin and even misclassified elements to be included, thus generating a greater bias but reducing the variance. The optimization of the regularization parameter was performed using an internal 5-fold cross-validation of the SVM function with the constructor option *probability* set to True.

The application of artificial neural networks (ANNs) in hyperspectral image classification for plant disease detection is widespread. In this study, we used the multilayer perceptron (MLP) classifiers, as they have shown to be effective in applications for the determination of behavioral patterns in high-dimensional data.

Multilayer perceptron (MLP) is a neural network consisting of several layers. A feedforward algorithm adjusts the weights and biases of each artificial neuron by applying an activation function. In the final layer, the loss function is computed, and backpropagation is initiated, which optimizes the weights and biases in order to cut down the loss function to zero using the gradient descent technique [30]. MLPs have been used as efficient classifiers, and it is documented that a two-hidden-layer MLP can form arbitrary classification regions, and a single-hidden-layer MLP can form single convex decision regions [31].

For our experiment, two multilayer perceptron (MLP) neural networks with backpropagation were designed, with one and two hidden layers, respectively.

The first artificial neural network (MLP 1) with a single hidden layer used the ReLu activation function, and in the output layer, a sigmoid function was applied. The sigmoid function outputs real values in the range of 0 to 1 that can be interpreted as the probability of infection. The number of hidden neurons was estimated with the pyramid rule, resulting in 22 neurons. The gradient optimizer was Adam [32], and the loss function was binary cross-entropy [33]. The training was performed by increasing the number of epochs from 100 to 1000 in increments of 50. Both the training and external validation results show that the network achieved the highest efficiency with 300 training epochs.

The second artificial neural network (MLP 2) with two hidden layers had 64 and 8 neurons, respectively, with the ReLu activation function. Again, the activation function in the output layer was the sigmoid function. Similar to MLP 1, the Adam gradient optimizer and the binary cross-entropy loss function were used. The model was trained by increasing the number of epochs from 100 to 1000. The maximum accuracy in validation was achieved with 350 training epochs.

## 3. Results

**Figure 3:**
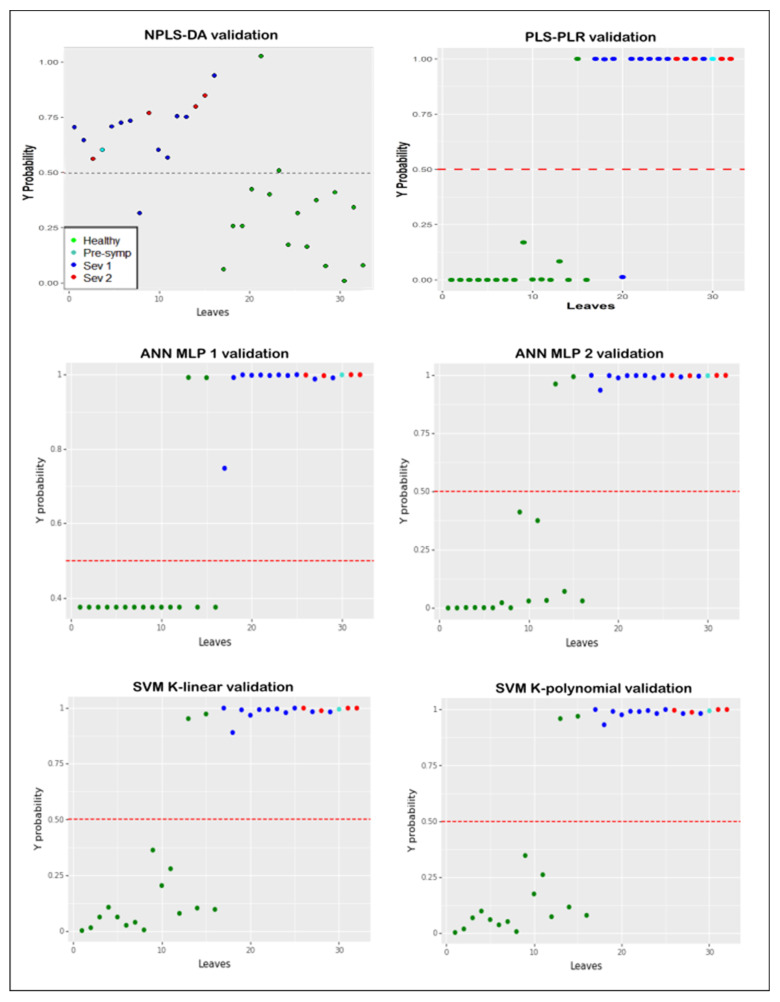
Estimated probability of infection from external validation of the models.

The models were able to predict the presence of the disease with high accuracy. The estimated infection probability was calculated on each leaf using the proposed models. The default decision threshold equaling 0.5 was used for the classification of the infected leaves. A score above that threshold indicated infection, and a score below indicated no infection. Figure 3 shows the estimated probability for the external validation of the models.

The prediction metrics were calculated directly from the confusion matrix results, i.e., true positive (TP), true negative (TN), true negative (TN), and false negative (FN). The evaluation of models’ performance was carried out by comparing the metrics of accuracy, precision, sensitivity, and F1.

Table 2 shows the training process results. Although the results of the two neural networks were similar, MLP 1 achieved the best results with 300 training epochs, and MLP 2 achieved it in 350 epochs; therefore, increasing the network complexity and computational load by including an additional hidden layer did not improve the results.

The results of the model validation tests were similar (Table 3). The PLS-PLR model obtained better precision, although its sensitivity was lower. NPLS-DA reported the lowest results.

The area under the ROC curve (AUC) measure provided evidence of the high discriminant ability of the built models. The AUC can be interpreted as the probability that a model classifies a true random positive higher than a false random positive. According to this metric, all the models had a very good discriminant ability.

The classification errors of the PLS-PLR, SVM, and MLP models were analyzed and are shown in Table 4.

PLS-PLR had errors in classifying the 15th and 20th leaves, while both the SVM and MLP models misclassified the 15th and 13th leaves.

**Figure 4:**
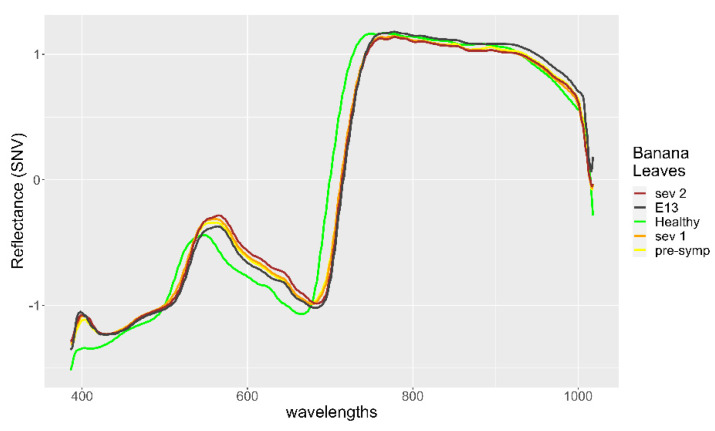
Spectral signature of 13th Leaf (E13 dark green color) compared with the spectral signature of healthy banana leaves and infected banana leaves.

When the spectral signatures of the misclassified leaves were compared with the average spectra of the healthy and diseased leaves, the similarities and differences that explained their erroneous classification could be observed. The spectrum of the 13th leaf shows that, in the range of yellow, orange, and red colors (560–780 nm), the reflectance values were close but lower than those of the presymptomatic diseased leaves, whereas in the near-infrared range (>780 mm), the reflectance was similar to that of healthy leaves reflectance. According to these characteristics, the spectrum of the 13th leaf was more similar to the spectrum of a healthy leaf (Figure 4).

**Figure 5:**
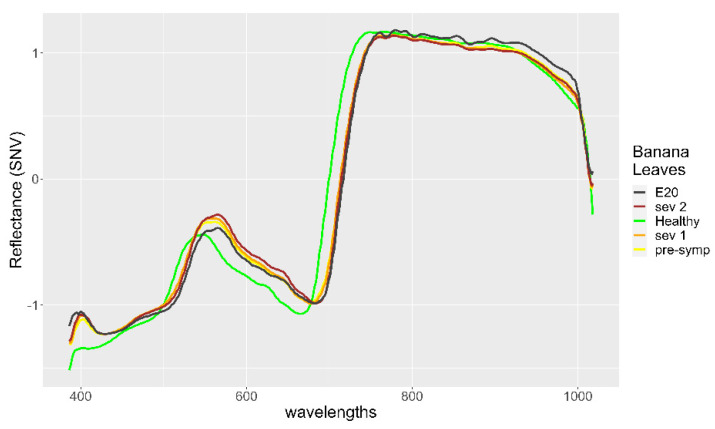
Spectral signature of 20th leaf (E20 black color) compared with the spectral signature of healthy banana leaves and infected banana leaves.

The 20th leaf presented, in the range of yellow, orange, and red colors (560–780 nm), reflectance values similar to those of the spectral signature of diseased leaves, while in the near-infrared range (>780 nm), the reflectance presented values even higher than those of the reflectance spectra of healthy leaves. Thus, the spectral signature of the 20th leaf was close to that of the diseased leaves with a very low level of infection (Figure 5).

**Figure 6:**
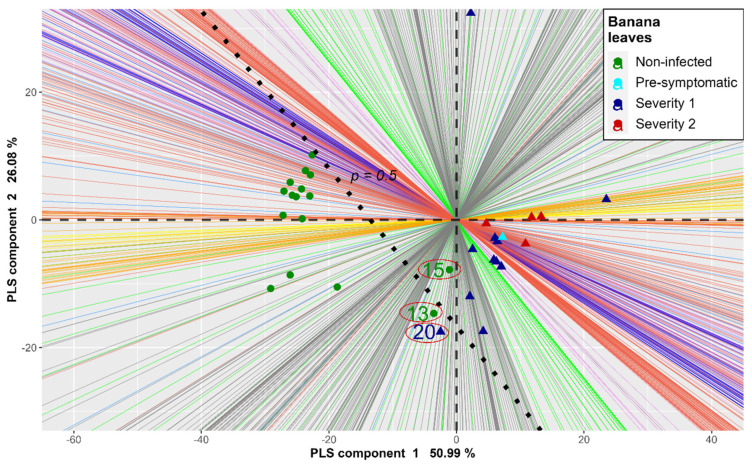
HS-Biplot of validation dataset highlighting misclassified leaves.

A final evaluation was performed using the HS-Biplot with the validation data (Figure 6). The HS-Biplot is a graph designed for hyperspectral image analysis. In this case, the leaves are represented as dots, and the wavelengths are presented as lines colored according to the electromagnetic band to which they belong [18]. Banana leaves are represented by points. Each wavelength is represented by straight lines colored according to electromagnetic spectrum colors. The oblique dotted line is the classification threshold and separates the healthy leaves from the infected ones. The green points show the control leaves, and turquoise, blue and red points represent the infected leaves at the initial stages.

It was observed that the 13th and 20th leaves were located close to the group of infected leaves with a greater influence of near-infrared wavelengths (gray lines) that places them toward the bottom of the graph very close to the classification threshold (0.5).

According to the HS-Biplot, both leaves were close to the group of diseased leaves, with a strong relationship in the waves of the near-infrared range and the green color, but had a low probability of infection, which places them below the threshold of classification, in the group of healthy leaves.

## 4. Discussion

Hyperspectral imaging generates a characteristic spectral signature with a general shape, position, and strength of the absorption bands that can be used to characterize different materials. The spectral signature of plants is determined by their physical–chemical attributes. Changes in pigments, structure, and water content modify the reflectance levels, which is manifested in the spectral signature, specifically, in the visible (VS 380–780 nm), near-infrared (NIR 780–1300 nm), and mid-infrared (NIR 780–1300 nm) spectral bands.

Several machine learning and deep learning methods offer high reliability in plant disease prediction using hyperspectral images, which was confirmed in this study. The obtained results showed a high predictive ability of PLS-PLR, NPLS-DA, SVM, and neural network (MLP) models, the evaluation of which was carried out by calculating the prediction metrics and the area under the ROC curve (AUC).

PLS is a technique widely used for the analysis of the datasets with high dimensionality and high collinearity, such as hyperspectral images. The learning principle is based on dimensionality reduction by maximizing the variability between the independent and dependent variables. PLS is a powerful tool for the analysis of high-dimensional data with minimum requirements in terms of measurement scales, sample sizes, and residual distributions. PLS-PLR has the advantage of using penalized logistic regression to relate the binary response to the PLS components, ensuring a better fit to the response. The prediction accuracy using LOOC cross-validation was 98%, and with validation data, the accuracy was 94%.

As input, NPLS-DA used a three-dimensional tensor obtained by calculating five statistical features calculated for each wavelength for each leaf. The output was calculated by applying the PLS1 algorithm and transformed by a logit function that delivered the probability of infection. NPLS took advantage of any multiway structure in the data, and the few parameters calculated gave it speed. This model does not include a regression constant, which reduces its predictive power in the case of binary responses. Nevertheless, it offered good results when the data points were linearly separable through the origin. The performance of this model was the lowest, but it achieved prediction results similar to those of the other methods (91% accuracy).

SVM is a widely used method in different classification and regression applications. The data transformation performed by the kernel function gives it great strength as a linear classifier with small or large datasets with separable and non-separable classes. Its algorithm is based on the inductive principle of structural risk minimization (SRM) that selects a marginal hyperplane with the largest margin that traverses the support vectors. Two SVM models were trained, one with a linear kernel and the other with polynomial kernels, applying variations in the regularization parameter. The developed models achieved the complete separation of the training data and, in the validation test, presented two errors with an accuracy equal to 94%.

MLP neural network is a gradient-based learning technique that focuses on the minimization of the loss function, which makes it a universal approximator. Two multilayer perceptron (MLP) models were developed, the first with one hidden layer and the second with two hidden layers, and were trained with the same dataset that was used to train the previous models. The training was started with 100 epochs and increased until the maximum accuracy was reached. The obtained results were equal to those of the SVM models. A wide and varied number of neural network architectures is available to approximate any complex decision surface that can classify high-dimensional data.

An analysis of the leaves misclassified by the PLS-PLR, SVM, and MLP models showed that, in this case, the classification performed by PLS-PLR was possibly the most accurate. PLS-PLR classification is more reliable for those applications in which the response is binary because it uses logistic regressions to calculate both the latent variables and the output, which allows the model to better fit the data.

## 5. Conclusions and Future Research Directions

The main contribution of this work is that we evaluated the predictive results of four machine learning and deep learning methods that have different ways of calculating the classification function to detect black sigatoka using hyperspectral images. This analysis can be generalized to other models with similar algorithms or learning principles according to the data conditions and research requirements.

The results obtained from the application of the PLS-PLR, NPLS-DA, SVM, and neural network methods in the classification of hyperspectral data confirmed their high discriminative and predictive capacity, offering a non-invasive method for the detection of plant diseases and assessing the efficacy of the chemical or biological control of the pathogens.

Hyperspectral sensor technologies have significantly improved in recent years, making a wide range of devices available to science that generate high-quality images, and a broad range of spatial and spectral resolutions, which can be analyzed using various machine learning methods. Close-range hyperspectral sensors can be used, in both stationary and airborne imaging system configurations connected to cloud and edge computing environments for real-time data processing.

Future research should investigate the characterization and detection of other diseases and environmental stresses that affect banana plants, which will allow the application and assessment of suitable control strategies.

## Figures and Tables

**Table 1:** Severity scale of banana leaves affected by black sigatoka disease (BLSD).

Stage	Symptoms
1	Yellow spots (<1 mm in diameter) on the abaxial leaf surfaces.
2	Red to brown streaks from 1 to 5 mm.
3	Red to brown streaks greater than 5 mm.
4	Brown elliptical streaks on the abaxial leaf surface and black streaks on the adaxial leaf surface.
5	The streak is fully black with a yellow halo and has spread to the abaxial leaf surface.
6	The center of the streak is light gray surrounded by a black ring and a yellow halo.

**Table 2:** Comparative table of models evaluation metrics in the training phase.

Training				
Models	Accuracy	Precision	Sensitivity	F1 Score
PLS-PLR ^1^	0.98	0.98	1	0.99
NPLS-DA	0.9	1	0.88	0.94
Linear SVM	1	1	1	1
Polynomial SVM	1	1	1	1
MLP 1	1	1	1	1
MLP 2	1	1	1	1

^1^ The PLS-PLR model was evaluated using the leave-one-out cross-validation (LOOCV) method. The SVM, MLP, and PLS-PLR models correctly classified all the training data, while the NPLS-DA model failed to separate the classes.

**Table 3:** Comparative table of models evaluation metrics in the validation phase.

Validation					
Models	Accuracy	Precision	Sensitivity	F1 Score	AUC
PLS-PLR	0.94	0.94	0.94	0.94	0.94
NPLS-DA	0.91	0.88	0.94	0.91	0.91
Linear SVM	0.94	0.89	1	0.94	0.94
Polynomial SVM	0.94	0.89	1	0.94	0.94
MLP 1	0.94	0.89	1	0.94	0.94
MLP 2	0.94	0.89	1	0.94	0.94

**Table 4:** Classification errors in external validation tests.

Classification Errors				
Model	Leaf Number	Probability	Prediction	Label
PLS-PLR	15	0.9999	Infected	healthy
	20	0.0061	healthy	Infected
Linear SVM	13	0.9548	Infected	healthy
	15	0.9757	Infected	healthy
MLP 1	13	0.9931	Infected	healthy
	15	0.9942	Infected	healthy

## Data Availability

The two datasets used in this study (i.e., the training dataset and validation dataset are available at: https://github.com/JUG2019/Sigatoka-detect (accessed on 21 August 2022).
